# Smartphone app aesthetics influence users' experience and performance

**DOI:** 10.3389/fpsyg.2023.1113842

**Published:** 2023-06-14

**Authors:** Sebastian A. C. Perrig, David Ueffing, Klaus Opwis, Florian Brühlmann

**Affiliations:** Human-Computer Interaction Research Group, Center for General Psychology and Methodology, Faculty of Psychology, University of Basel, Basel, Switzerland

**Keywords:** aesthetics, performance, usability, mobile devices, smartphones, User Experience (UX)

## Abstract

Past research has demonstrated that aesthetics affect users' experiences in various ways. However, there is little research on the impact of interface aesthetics on user performance in a smartphone app context. The present paper addresses this research gap using an online experiment (*N* = 281). Two variants of the same web app were created and manipulated in their aesthetics. Participants were randomly assigned to either variant and asked to explore the app before answering questions concerning the app's content. Results showed a significant positive effect of aesthetics on perceived usability and aesthetics. Furthermore, results point toward a positive impact of interface aesthetics on performance (i.e., the number of questions answered correctly). Thus, results indicate that a visually appealing smartphone web app increases users' subjective experience and objective performance compared to an unaesthetic app. This suggests that user interface aesthetics impact users' experiences and provide stakeholders with quantifiable value and competitive advantage.

## 1. Introduction

Smartphone use is developing rapidly worldwide. While there were 2.49 billion active smartphone users in 2016, this number has risen to 3.6 billion in the following 4 years, and by 2024, 4.5 billion active users are expected (Tenzer, 2022). Furthermore, 54.97% of all website visits worldwide in 2021 were made via smartphones (Statista Research Department, 2022) and smartphones are expected to replace computers in certain areas of daily life (Anderson, 2019). It is, therefore, not surprising that many software developers frequently develop mobile device applications (apps) or port their computer programs to them. A shift in focus by developers and businesses from computer programs to apps has resulted in the ability to perform almost any daily task with an app, ranging from contacting friends to banking transactions. There appears to be an app for each activity, or a whole market of specific apps for each task, resulting in a competitive market where users can choose between various alternatives. Given the omnipresence of smartphone apps in private and professional life, the question arises as to what makes a smartphone app successful in such a highly competitive market. Several indications point to aesthetics, which has a multi-layered influence on people's perceptions. An example of this is the influence of the aesthetics of an app on users' subjective evaluation, which can take place within fractions of a second (Guo et al., 2020). It is thus unsurprising that in the developer community and human-computer interaction (HCI) field, more and more attention is being paid to aesthetics (Tractinsky and Hassenzahl, 2005). Several studies have shown a positive effect of aesthetics on subjective perception and the resulting reactions (De Angeli et al., 2006; Thüring and Mahlke, 2007; Douneva et al., 2016). Furthermore, some researchers have already demonstrated that aesthetics positively affects performance in various contexts (Salimun et al., 2010; Sonderegger and Sauer, 2010; Reppa and McDougall, 2015). However, to our knowledge, there is still limited empirical investigation into the effects of aesthetics within a smartphone app context, despite the growing importance of the mobile device market. It thus remains unclear to what extent past findings concerning the impact of aesthetics on the users' experiences and performance can also be found within the smartphone device context. The present study thus investigated the effect of smartphone app aesthetics on users' subjective perception of aesthetics and usability and users' performance with an experimental study to address this research gap.

## 2. Related work

### 2.1. A brief excursion into the world of apps

Mobile interfaces differ substantially from desktop websites (Nielsen and Budiu, 2013). For example, given the smaller screen size, less information can be displayed simultaneously, and while exact clicking on smaller targets is possible with precise mouse movements on desktop websites, less precision is possible on smaller smartphone touch screens (Nielsen and Budiu, 2013). Thus, while we might assume that results from a desktop setting also apply to a smartphone context, we can only be sure once an empirical investigation is conducted. In addition, past research has already shown that non-smartphone mobile devices differ from desktop websites concerning the effect of aesthetics on performance (Thielsch et al., 2019b). Smartphones, however, which differ from past mobile devices (e.g., because of touchscreens), have not yet been studied in this respect. Further, Groth and Haslwanter (2015) found significant differences in perceived usability and user experience between desktop computers and smartphones, while Nielsen and Budiu (2013) found lower e-commerce conversion rates for mobile phones in contrast to desktop computers, and Zhu et al. (2020) showed that written user reviews differ between mobile and desktop devices in several aspects (e.g., fewer words and more pictures). Thus, past research has shown that results from a desktop setting can differ from those found in a mobile context, but the effect of aesthetics on performance still needs to be determined for smartphone apps.

Although the term *app* is used frequently, it does not always imply the same thing. According to the Merriam-Webster Dictionary, the term *application* refers to “a program (such as a word processor or a spreadsheet) that performs a particular task or set of tasks.”^1^ In contrast, the term *app* describes “an application designed for a mobile device (such as a smartphone).”^2^ A further distinction is made between native and web apps (Jobe, 2013). A native app is downloaded from a store and permanently installed on the smartphone, with a separate app programmed for each platform (El-Kassas et al., 2017). On the other hand, a web app is a particular form of an interactive website that behaves like a conventional application but does not have to be installed on a smartphone, which is a great advantage of web apps (Jobe, 2013). In the case of mobile versions of a website, the term *generic mobile web application* refers to versions of a website either developed for a mobile context or adapted through responsive design (Jobe, 2013). Web apps can be used across platforms and do not require custom programming for each operating system. In addition, developers can distribute updates to all users faster and more efficiently, as there is no need to trigger a manual update process as with native apps (Liu et al., 2015). Studies have also shown that web apps perform better than native apps under certain conditions (Jobe, 2013; Liu et al., 2015; Ma et al., 2017). Large companies increasingly recognize these advantages of web apps over native apps to better reach and support users. While Google is moving forward with plans to foster web apps,^3^ Microsoft released its game streaming platform *Xbox Cloud Gaming* as a web app for multiple platforms.^4^ Similarly, Apple allows developers to launch applications as web apps (Apple Pty Ltd., 2021). Experts, therefore, agree that web apps will increasingly be found on the market in the future, offering an excellent alternative to native apps (Ater, 2017).

### 2.2. Aesthetics in HCI

Initiated by works such as Kurosu and Kashimura (1995) or Tractinsky et al. (2000), aesthetics has been extensively investigated within the field of HCI. Past research provided evidence that visually appealing websites are perceived as more trustworthy (Lindgaard et al., 2011) and that user purchase intent increases with more appealing systems (Hausman and Siekpe, 2009), as do satisfaction (Lindgaard, 2007) and preference (Lee and Koubek, 2010). From a psychological point of view, aesthetics appear to satisfy basic human needs of enjoyment and wellbeing (Postrel, 2004). Furthermore, when it comes to self-expression, users can express their individuality by personalizing interfaces or lock screens, allowing them to differentiate themselves from others (Hassenzahl, 2018). Lee and Koubek (2010) further showed that users initially evaluate an interactive system significantly based on its aesthetic impression, while Wiecek et al. (2019) found that product aesthetics (e.g., smartphone cases) had a positive effect on usage intensity while deterring users from switching to different products. Over the past two decades, such promising research results have enabled designers and the HCI community to move away from initial concerns by some (e.g., Andre and Wickens, 1995) that aesthetic design interferes with work objectives. Aesthetics is now a widely recognized “must-have” factor that gets a great deal of attention when developing systems (Thielsch et al., 2014).

#### 2.2.1. Perceived visual aesthetics

Moshagen and Thielsch (2010) defined aesthetics "as an immediate pleasurable subjective experience that is directed toward an object and not mediated by intervening reasoning" (p. 690). According to Lavie and Tractinsky (2004), aesthetics can be separated into classic and expressive aesthetics. Classic aesthetics refers to clean, pleasant, and symmetrical attributes, while expressive aesthetics refers to characteristics such as creative, original, and sophisticated. Moshagen and Thielsch (2010) further argued that the construct of visual aesthetics is represented by four facets: simplicity, diversity, colorfulness, and craftsmanship. Simplicity describes concepts like unity or homogeneity, while diversity represents aspects such as novelty and creativity. Simplicity correlates highly with classic, and diversity correlates highly with expressive aesthetics of Lavie and Tractinsky (2004). Colorfulness considers aspects such as the placement and combination of colors. Finally, craftsmanship reflects whether the product has a harmonious design and uses modern technologies. Given that multiple studies have investigated this conceptualization of aesthetics (e.g., Moshagen and Thielsch, 2010, 2013) where it has proven itself useful, this paper will follow this definition by Moshagen and Thielsch (2010).

#### 2.2.2. Objective facets of aesthetics

Examining aesthetics raises the question of how products can be objectively manipulated to realize different aesthetic impressions. Various studies have shown two salient characteristics, complexity and symmetry, to strongly influence the perception of websites (Bauerly and Liu, 2008; Lai et al., 2010; Tuch et al., 2010; Bi et al., 2011; Seckler et al., 2015). Moreover, they proved to be some of the most distinctive design features upon initial observation (Leder et al., 2004). Bauerly and Liu (2008) postulated that symmetry helps viewers structure content by creating regular and meaningful forms. Moreover, in Seckler et al. (2015), symmetry was the biggest influencing factor on the subjective overall aesthetic perception. In contrast, complexity is more challenging to define (Xing and Manning, 2005). Nevertheless, several studies described visual complexity by the quantity of objects, clutter, openness, symmetry, organization, and variety of colors (Olivia et al., 2004; Michailidou et al., 2008; Riegler and Holzmann, 2018). Based on this definition, multiple HCI studies provided evidence for a negative linear correlation between visual complexity and aesthetic perception, implying that higher complexity leads to lower aesthetic ratings (Michailidou et al., 2008; Tuch et al., 2012a; Seckler et al., 2015).

Besides complexity and symmetry, color was repeatedly shown to be among the most striking design features at first glance (Cyr et al., 2010; Reinecke et al., 2013). In the context of HCI, color is frequently represented by the Hue-Saturation-Brightness (HSB) model, according to which color is composed of three parts: hue, saturation, and brightness (Smith, 1978). Hue is defined as a pure, spectral color such as blue, red, or yellow. In various studies, blue and gray websites were rated as the most attractive and yellow and purple as the least attractive ones (Cyr et al., 2010; Seckler et al., 2015). Comparable results have also been found in studies not related to HCI (Fortmann-Roe, 2013; Palmer et al., 2013; Oyibo and Vassileva, 2020). Saturation, the second aspect of the HSB model, describes the intensity of the color, which has not been extensively researched to date (Seckler et al., 2015). Nevertheless, there is an indication that western adults generally prefer higher saturated websites (Palmer and Schloss, 2010; Lindgaard et al., 2011; Seckler et al., 2015). Brightness, the last aspect, describes the perceived luminance of a color. As with saturation, there is little scientific evidence on the effects of brightness (Seckler et al., 2015). However, some evidence indicates that websites with high background luminance are rated as the most beautiful (Palmer and Schloss, 2010; Lindgaard et al., 2011).

#### 2.2.3. Effects of aesthetics on usability

The positive effect of aesthetics on various subjective aspects of users' experiences, such as preferences and trust (Moshagen and Thielsch, 2010), user satisfaction (Tractinsky et al., 2000; Lavie and Tractinsky, 2004; Tseng and Lee, 2019), or joy of use (Lingelbach et al., 2022) has already been demonstrated and widely researched. Another frequently studied subject is the effect of aesthetics on usability. The International Organization for Standardization (2018) defines *system usability* as “the extent to which a system, product or service can be used by specified users to achieve specified goals with effectiveness, efficiency and satisfaction in a specified context of use.” In research, a distinction is made between subjective and objective usability. Subjective usability concerns users perception and attitudes regarding a system, while measures of objective usability evaluate a systems properties not dependent on a persons perception (Hornbæk, 2006). Researchers, therefore, addressed the question of what subjectively perceived usability depends on. Several studies have found a robust effect of aesthetics on subjective usability, showing that users working with a more attractive system rated it as more usable than users of a less attractive one (Moshagen et al., 2009; Sonderegger and Sauer, 2010; Sonderegger et al., 2014; Gu et al., 2016; Minge and Thüring, 2018; Otten et al., 2020; Schrepp et al., 2021).

### 2.3. Aesthetics and performance—current state of research

Prompted by aesthetics' effects on users' subjective experiences, the question of whether visual aesthetics also influence an objective construct such as performance arose. In this paper, *performance* is defined in line with Thielsch et al. (2019b) as “an objectively measurable outcome of a user's interplay with a website, software or other interactive system” (p. 200). While there is initial evidence for an effect of aesthetics on performance, it is not yet clear whether users only believe that they perform better with a more aesthetic application or whether there is an objectively measurable change in performance. Research results thus far are ambivalent (Thielsch et al., 2019b). Some studies support a performance improvement when interacting with an aesthetically more appealing interface (Sonderegger and Sauer, 2010; Douneva et al., 2016; Baughan et al., 2020; Reppa et al., 2021), whereas others show a contrary effect (Sauer and Sonderegger, 2011; Sonderegger et al., 2014). In addition, several studies could not show any significant effect (Douneva et al., 2015; Gu et al., 2016; Thielsch et al., 2019a). Given these contradictory findings, various explanations have been made to understand aesthetics' effect on performance, summarized in the following section.

#### 2.3.1. Theoretical considerations

Szabo and Kanuka (1999) postulated that good design improves performance by reducing cognitive processing effort. This reduced effort is achieved because good design enables faster recognition of visual objects. In this regard, good design is implemented through low complexity and higher coherence, promoting the automatic processing of information. Bad design, on the other hand, provokes more inefficient, manual processing (Szabo and Kanuka, 1999). Inspired by this idea, various researchers have discussed attentional effects of aesthetic design (e.g., Reppa et al., 2008). In this context, additional cognitive effects of website perception have been debated, such as visual complexity and prototypicality, bottom-up perception processes, and mental models (Tuch et al., 2009; Douneva et al., 2016).

Tractinsky et al. (2000) took the idea of the halo effect from Psychology^5^ and postulated that “what is beautiful is usable,” arguing that the user infers from the aesthetic design to other parts of the application. For example, due to the halo effect, the user initially perceives an application as aesthetic and concludes from this judgment alone that the application has good functionality. Some studies provided evidence for this assumption (Lavie and Tractinsky, 2004; Hartmann et al., 2008; Quinn and Tran, 2010), while others found a reversed effect under certain conditions (Tuch et al., 2012b).

Sonderegger and Sauer (2010) argued that aesthetic design puts users at ease or in a kind of "flow state" (Csikszentmihalhi, 1997). In this state, users perceive the tasks given to them as congruent with their abilities, leading to faster processing and increased motivation when using a system, consequently increasing performance. This is especially the case in a work context. They further claimed that users focus on a design that is subjectively perceived as beautiful and then "lose themselves" in it, leading to more inefficient processing and, thus, lower performance. Users in such situations are no longer fully focused on the task but try to prolong the pleasant experience of interacting with the appealing design. This "prolongation of joyful experience" occurs more often in leisure tasks, focusing on fun and enjoyment rather than performance (Sonderegger and Sauer, 2010; Sonderegger et al., 2014).

Overall, there are few systematic studies on these explanatory concepts (Thielsch et al., 2019b), and results on the relationship between aesthetics and performance are often contradictory. Thielsch et al. (2019b) have taken this as an occasion to conduct a meta-analysis. Results revealed a small, positive effect of interface aesthetics on user performance (*g* = 0.12). Moreover, a complementary finding was that more aesthetically pleasing variants significantly impact user performance, especially when interacting with mobile devices and software applications. However, the studies and data available to date are far from adequate, leading the authors to formulate a call to action for more substantiated research.

### 2.4. Study goals

As Thielsch et al. (2019b) suggested in their meta-analysis, aesthetics influence user performance in the context of digital products. However, their results should be regarded with caution, as there were several challenges with the included studies. First, the authors emphasized that there are still too few high-quality publications that address the relationship between aesthetics and performance. Therefore, further research is essential to understand aesthetics' effect on user performance better. Furthermore, previous studies have primarily focused on computer applications. However, smartphones, with their smaller displays and on-the-go use, have unique requirements and strengths (Adepu and Adler, 2016). Thus, previous findings on computer interfaces may not directly apply to smartphone interfaces and apps. Research addressing mobile devices to date mainly focused on the external appearance of the device as an aesthetic manipulation (e.g., Sonderegger and Sauer, 2010; Sonderegger et al., 2014; Minge and Thüring, 2018). Thus, there is a lack of studies centering on mobile devices' interfaces.

The present work addresses these issues by focusing solely on an app's user interface rather than a smartphone's exterior design. The specific device used by participants was not considered as long as participants used a smartphone device to access the online study. Specifically, this study examined the impact of an app's interface aesthetics on user performance during use. To investigate aesthetics, we employed the definition of Moshagen and Thielsch (2010, 2013). Perceived usability and aesthetics were measured using two validated survey scales. A set of self-developed knowledge questions related to the app's content filled out post-interaction were used to quantify performance. Overall, this study aimed to address the current research gap by investigating the effect of interface aesthetics on performance in the context of mobile devices. The results promote a deeper understanding of user performance and behavior in the context of smartphone use and the influence of aesthetics on such interactions.

#### 2.4.1. Research hypotheses

We derived the following three research hypotheses based on the study goals and previous research described above:

- H1: Concerning perceived usability, users of the aesthetically pleasing variant of the app will exhibit higher levels of subjective usability than users of the unaesthetic one.
- H2: Concerning task completion time, users of the aesthetic variant of the app will complete tasks related to the app content faster than users of the unaesthetic variant.
- H3: Considering task performance, reflected in a performance score, users interacting with the aesthetic variant of an app will have a higher performance score, compared to those interacting with the unaesthetic variant.

## 3. Materials and methods

To achieve our research goals, we conducted a between-subjects design online experiment. Participants interacted with one of two variants of a fictitious event agency's web app. The two variants of the app were manipulated in terms of aesthetics to investigate a possible relationship between the app's aesthetics and the user's performance and experience during the interaction.

### 3.1. Sample

We recruited an initial sample of 387 participants over Amazon Mechanical Turk (MTurk),^6^ out of which 344 completed the online experiment. Ethical review and approval was not required for the study in accordance with the local legislation and institutional requirements. The participants provided their written informed consent to participate in this study. Only workers located in the United States of America with a human-intelligence-task approval of 95% and at least 100 approved tasks were allowed to participate in the experiment. For data cleaning purposes, we imposed several criteria on the sample. First, all subjects who indicated a visual or color impairment were removed (*n* = 22) because participants had to perceive and evaluate aesthetics manipulated by color, among other things. Following recommendations by Brühlmann et al. (2020), we removed one participant for failing to correctly answer an attention check item (Meade and Craig, 2012; Curran, 2016), and one respondent because they self-reported that their data should not be used due to insufficient quality (Meade and Craig, 2012). Seven participants were removed due to interruptions while answering the survey. Furthermore, we removed five participants for responding to the Visual Aesthetics of Websites Inventory (VisAWI, Moshagen and Thielsch, 2010) and Usability Metric for User Experience (UMUX, Finstad, 2010) too quickly (following Huang et al., 2012) and 20 participants who took too long to answer the survey (outliers concerning response time based on the interquartile range). Finally, we removed seven participants with a suspicious amount of the same answers for the VisAWI and UMUX, indicating that they ignored the reverse-coded answers (i.e., same answers not only across all positively formulated items but also for reversed items). After data cleaning, a final sample of 281 complete responses remained (aesthetic condition = 139, unaesthetic condition = 142). Participants self-reported an average age of 35.39 years [standard deviation (*SD*) = 9.77, *range* = 18 − 70] and 137 participants identified as female (male = 135, non-binary = 5, preferred not to answer = 4).

### 3.2. Materials and experimental manipulations

**Figure 1:**
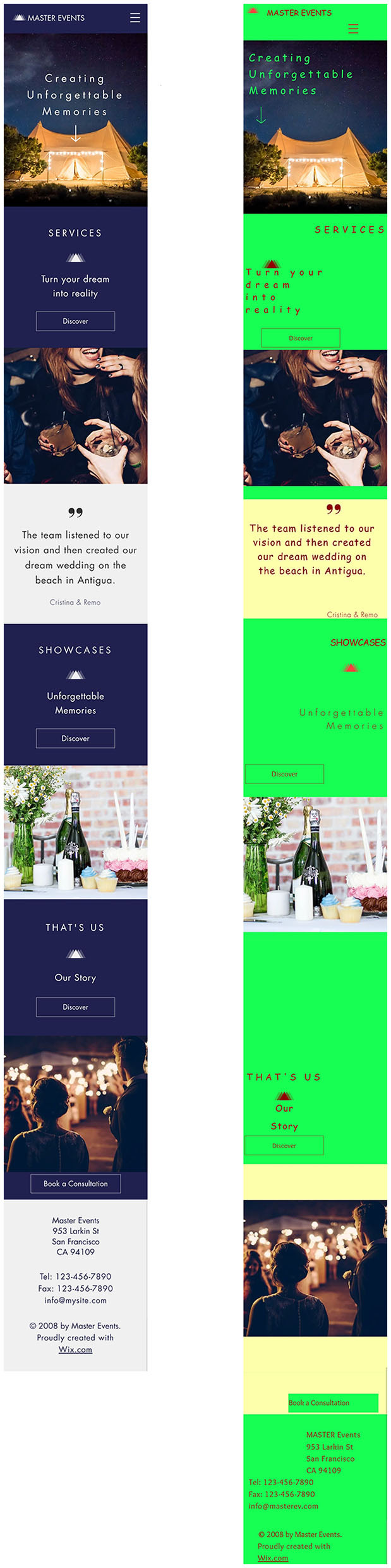
The two final variants of the web app used as stimuli in this study. Shown is the landing page of the aesthetic **(left)** and the unaesthetic **(right)** implementation. Images used from Unsplash. Note that the first image depicted in the screenshots was replaced with a comparable image for this publication due to copyright.

To reveal possible effects of aesthetics on performance, following the findings of Thielsch et al. (2019b), different variants of the same app were created and manipulated to be either as aesthetically pleasing or as unaesthetic as possible. In line with past research, we opted for manipulating aesthetics as much as possible to avoid problems caused by weak manipulation (Thielsch et al., 2019a). For the final study, two variants of the same app (Figure 1) were developed using the free website development platform Wix.^7^ Care was taken to keep all aspects of the app not related directly to aesthetics the same, including avoiding strong manipulations of system usability. Therefore, we purposefully refrained from altering system properties related to usability in past research, such as manipulation of the information architecture (e.g., menu labels as in Tuch et al., 2012b), menu structure (as in Minge and Thüring, 2018) or page response time (e.g., system delay as in Tractinsky et al., 2000). Aesthetics was thus manipulated in line with past research by manipulating symmetry and color combinations (e.g., Minge and Thüring, 2018) or changing the website structure, color, and fonts while keeping the content constant (as in Iten et al., 2018). In addition, we considered the Web Content Accessibility Guidelines (Accessibility Guidelines Working Group, 2018) to keep both variants as comparable as possible. For example, the contrast ratios of the elements for both variants were always at least level AA according to the guidelines. In general, the base variant of the app before manipulation was designed to be as realistic as possible. In addition, efforts were made to maximize the difference in aesthetics between the two final variants of the app. The following subsections describe the development of the two app variants in more detail.

#### 3.2.1. Initial stimuli design

Feedback was gathered from a team of experts during various stages of the design process to ensure a realistic app design. Specifically, four user interface and user experience designers were consulted, and their feedback was incorporated into the development of the apps. These experts contributed their expertise in aesthetic and user-centered software design in individual discussions. This way, efforts were made to develop a realistic and well-executed initial app. This base app was then manipulated regarding aesthetics, based on the conceptualization of aesthetics by Moshagen and Thielsch (2010), to create seven different app variants. For creating these app variants, three aspects of aesthetics were varied: color, complexity, and symmetry. Different color combinations were used, shown to be perceived by users as particularly aesthetic or unaesthetic in past research (Seckler et al., 2015). Different amounts of colors were included in the color scheme of the respective app variant to manipulate complexity. Furthermore, the number of fonts was varied to alter the consistency of the app variants, and thus the complexity of the overall appearance (Thielsch et al., 2019a). Symmetry was manipulated mainly by deviating from the central vertical axis of the screen.

#### 3.2.2. Preliminary stimuli evaluation

The seven initial app variants were compared in a preliminary evaluation to select the variants with the highest and lowest aesthetics ratings as stimuli for the main study. A total of 12 HCI researchers (master's and Ph.D. students enrolled in the HCI program at the authors' university) rated screenshots for each of the seven app variants using the four-item short version of the VisAWI, the VisAWI-S (German version, Moshagen and Thielsch, 2013).^8^ In addition, participants answered an ordering question that asked for all variants to be sorted from highest to lowest aesthetics. The VisAWI-S score of the app variant rated highest [mean (*M*) = 5.23, *SD* = 1.23] exceeded the cut-off of 4.5 for an aesthetic design by Hirschfeld and Thielsch (2015) and differed clearly from the variant rated lowest (*M* = 2.25, *SD* = 1.02). Ratings from the ordering question were also consistent with the VisAWI-S ratings. Furthermore, we performed a one-way analysis of variance (ANOVA) to compare the app variants' effect on the VisAWI-S score. Results revealed a statistically significant difference between at least two variants [*F*_(6, 77)_ = 14.10, *p* < 0.0001, η^2^ = 0.52]. Because the VisAWI-S score was not normally distributed, we further calculated a Kruskal-Wallis rank sum test, which also showed a significant difference [χ^2^(6) = 44.07, *p* < 0.0001]. Finally, Tukey's Honest Significant Difference Test for multiple comparisons showed that the mean value was significantly different between the app variant rated highest and the variant rated lowest [*p* < 0.0001, difference in means = 2.98, 95% CI (1.59, 4.37)].

#### 3.2.3. Final stimuli used

Figure 1 shows the two final app variants used in the main experiment. For the aesthetic variant, based on findings by Seckler et al. (2015), only the colors blue and gray were used (see Supplementary material for exact color codes).^9^ In addition, we used only one font type (*Futura*) across the app. Due to the small number of colors and only one font, we considered this condition of low complexity. We kept symmetry at a maximum throughout the app. Every element was aligned around a vertical, central axis, and care was taken to ensure that each element occupied approximately the same amount of space. In the unaesthetic variant, six different color variations were chosen based on Seckler et al. (2015), including three shades of red. Furthermore, we used three different fonts across the app (*Comic Sans MS, Overlock*, and *Futura Light*). Thus, the complexity in this app variant was arguably higher than in the aesthetic variant. Wherever possible, symmetry was purposefully disregarded. Emphasis was placed on arranging the various surface objects as asymmetrically as possible so that no symmetry or pattern could be discerned.

### 3.3. Measurements

Two validated self-reported survey scales from previous research were used for data collection alongside two indicators of performance (performance score, performance time). Before interpreting the data, we investigated the scales' reliability and validity to ensure the quality of our measurements, which should always be done whenever scales are used with a new sample (Furr, 2011). The scale used to measure aesthetics was not previously validated in its English version but only in German with German-speaking participants (Abbas et al., 2022). The scale's quality in English was thus unclear. In addition, both scales were developed with non-mobile devices, so we wanted to ensure sufficient scale quality in our context before interpreting the results. Reliability was investigated using two measures of internal consistency, coefficients α (Cronbach, 1951) and ω (McDonald, 1999). Regarding validity, we investigated the structure of all survey scales using confirmatory and exploratory factor analysis. The essential parts of these investigations are reported as part of the following subsections, while full details are provided on the Open Science Framework (OSF).^10^

#### 3.3.1. Perceived visual aesthetics: the VisAWI

The VisAWI (Moshagen and Thielsch, 2010) was used to measure the perceived visual aesthetics of the app. The VisAWI is a self-reported survey scale comprising 18 items (including eight negatively formulated items) distributed over four subscales: *Simplicity, diversity, colorfulness*, and *craftsmanship*. Ratings were made on a 7-point Likert-type scale ranging from 1 (strongly disagree) to 7 (strongly agree). Scale values for the subscales were formed by calculating means across items for each subscale, while the overall score was calculated by adding up the four subscale values and dividing them by four (Thielsch and Moshagen, 2015). The internal consistency of the VisAWI total score was excellent according to George and Mallery (2019) [α = 0.96, 95% CI (0.95, 0.97), ω_*h*_ = 0.95, 95% CI (0.93, 0.96)], and between good and excellent for the four subscales: *Simplicity* with five items [α = 0.86, 95% CI (0.83, 0.89), ω = 0.86, 95% CI (0.82, 0.88)], *diversity* with five items [α = 0.87, 95% CI (0.84, 0.90), ω = 0.88, 95% CI (0.84, 0.90)], *colorfulness* with four items [α = 0.91, 95% CI (0.89, 0.93), ω = 0.91, 95% CI (0.89, 0.93)], and *craftsmanship* with four items [α = 0.87, 95% CI (0.84, 0.90), ω = 0.87, 95% CI (0.83, 0.90)].

The theoretical structure of the VisAWI was assessed with a Confirmatory Factor Analysis (CFA) using the lavaan package for R (version 0.6-11, Rosseel, 2012). We examined the proposed four-factor model (i.e., simplicity, diversity, colorfulness, and craftsmanship), including a higher-order factor for overall aesthetics. All items were specified to load on their designated factor, and the first item's loading was constrained to one. Multivariate normality was not given (Henze-Zirkler Test = 2.44, *p* < 0.0001); therefore, a robust maximum likelihood estimation method with Huber-White standard errors and a Yuan-Bentler based test statistic was used. Results of the CFA including all 18 items suggested that the proposed model does not adequately fit the data [χ^2^(131) = 674.47, *p* < 0.0001, *CFI* = 0.84, *SRMR* = 0.08, *RMSEA* = 0.14].^11^ We consequently performed an exploratory factor analysis (EFA) for the VisAWI data, which suggested a two-factor solution. Factor one consisted of the ten positively formulated items of the VisAWI, while the eight negatively formulated items mostly loaded onto the second factor or cross-loaded onto both. It thus appeared that the item wording (positive or negative) influenced the scale's factor structure. Such a phenomenon has been reported for other scales, including the System Usability Scale (SUS) (Brooke, 1996). In the case of the SUS, Lewis and Sauro (2017) recommended treating the scale as a unidimensional measure due to the limited interest that comes with a distinction based on negative/positive item tone. Following this example, we decided to stick with a one-factor solution for the VisAWI as an indicator of *perceived aesthetics* because a distinction between the two factors was theoretically non-sensible. We further refrained from interpreting the four sub-scales of the VisAWI. A one-factor EFA showed that this one-factor solution explained 60% of variance, while a one-factor CFA indicated a comparable fit to the original model [χ^2^(135) = 728.46, *p* < 0.0001, *CFI* = 0.82, *SRMR* = 0.08, *RMSEA* = 0.15].

#### 3.3.2. Perceived usability: the UMUX

The UMUX (Finstad, 2010) was used to measure participants' perceived usability of the respective app variant. The UMUX consists of four items rated using a 7-point Likert-type scale ranging from 1 (strongly disagree) to 7 (strongly agree). The even items of the scale were reversed before scoring, after which responses were transformed into a score ranging from 0 to 100. The survey scale exhibited acceptable internal consistency according to George and Mallery (2019) [α = 0.81, 95% CI (0.76, 0.85), ω = 0.79, 95% CI (0.67, 0.83)].

As with the VisAWI, we performed a CFA to assess the factor structure of the UMUX data as an indicator of scale validity. All four items of the UMUX were specified to load onto one factor, and the loading of the first item was constrained to one. Multivariate normality was again not given (Henze-Zirkler Test = 16.77, *p* < 0.0001); therefore, the same robust maximum likelihood estimation method was used. Results of the CFA suggested an inadequate fit of the proposed model to the data [χ^2^(2) = 87.52, *p* < 0.0001, *CFI* = 0.73, *SRMR* = 0.14, *RMSEA* = 0.50]. As with the VisAWI, we thus performed an EFA for the UMUX data. The EFA suggested a two-factor solution, with one factor for the two positively formulated items and a second for the two negative items. Following the same logic as with the VisAWI, we decided to adhere to the originally proposed one-factor solution for the UMUX, representing *perceived usability*, able to explain 52% of variance (according to a one-factor EFA).

#### 3.3.3. Dependent variable: performance score

Following prior research (Moshagen et al., 2009; Sonderegger et al., 2014; Thielsch et al., 2019b), performance was measured both by a *performance score* using six content-related questions and the task completion time for answering these six questions, hereafter referred to as *performance time*. A high performance thus meant answering as many questions of the information foraging task correctly and having a short performance time.

Participants were asked to answer six questions targeting the app's content to assess the performance score (e.g., “Since when has the Master Events agency been in business?"). These questions were developed in iterative discussions with members of the authors' research group. The exact questions are documented in the Supplementary tables and figures on OSF. Each question asked for specific details about the fictional event agency and offered four answer choices, of which only one was correct. Participants had to select the correct answer in each case. Answers to the questions were presented in randomized order to avoid any order effects. One point was awarded for each correct answer, resulting in a minimum of 0 and a maximum of 6 points per participant. The score obtained represented the performance score. The average performance score achieved by participants was 5.10 points (*SD* = 1.42, *range* = 0 − 6). Internal consistency for the six questions was acceptable according to George and Mallery (2019) [α = 0.76, 95% CI (0.70, 0.82), ω = 0.77, 95% CI (0.71, 0.83)]. In addition, each item's difficulty and item discrimination was considered to evaluate the performance score further. The mean value across all respondents for each item served as item difficulty, indicating how many participants answered the item correctly. Item difficulty ranged from 0.74 to 0.95, indicating that all items had a reasonable and comparable level of difficulty and could thus be mastered by conscientious participants, although the items were arguably on the easier side. This is comparable to past research, where most participants were able to complete the performance tasks [82% successful task completion in Sonderegger et al. (2014) and difficulty of 0.76 in Thielsch et al. (2019a)]. Item discriminatory power was calculated from the correlation of the item with the score across the other five performance questions (corrected item-total correlation). Values ranged from 0.52 to 0.66, all within the ideal range of between 0.40 and 0.70 (Moosbrugger and Kelava, 2000) and above the lowest acceptable discriminatory power of 0.30 according to Borg and Groenen (2005). The Supplementary tables and figures on OSF contain all values for item difficulty and discriminatory power.

Finally, we conducted a CFA to assess the factor structure of the performance items. All six performance questions were specified to load onto one factor, and the loading of the first item was constrained to one. The same robust maximum likelihood estimation method was used as multivariate normality was again not given (Henze-Zirkler Test = 90.55, *p* < 0.0001). Results of the CFA mostly suggested that the proposed model adequately fits the data [χ^2^(9) = 14.91, *p* = 0.09, *CFI* = 0.97, *SRMR* = 0.04, *RMSEA* = 0.07]. Only the RMSEA was slightly above the desired value of < 0.06 (Hu and Bentler, 1999).

#### 3.3.4. Dependent variable: performance time

Performance time was collected automatically by the online survey tool. The average time needed by participants to answer all six questions was 2.59 minutes (*SD* = 2.15 minutes, *range* = 0.22 − 14.07 minutes).

### 3.4. Procedure

The online study featured a between-subjects design with manipulated app aesthetics (high vs. low). Participants were randomly assigned to one of two conditions, resulting in two groups of comparable size (high aesthetics: *n* = 139; low aesthetics: *n* = 142). The two groups did not differ significantly regarding the demographic variables age [*F*_(1,279)_ = 0.34, *p* = 0.56, η^2^ < 0.01.] and gender [χ^2^(3) = 3.30, *p* = 0.35, Cramer's *V* = 0.11]. The study consisted of four phases and took participants on average 8.94 minutes to complete (*SD* = 3.59 min, *range* = 2.05 − 18.98 minutes). Data collection for the study was conducted using the online survey tool Unipark.^12^

In the study's first phase, the survey platform automatically checked if participants accessed the study using a mobile device. Access from other device types was denied. Once participants could access the site, they were presented with an introduction briefly explaining the study's purpose. Here, participants were informed about the study characteristics (duration of data storage, anonymity, and compensation) and provided informed consent. Afterward, demographic data (age and gender) was collected. Participants had to be at least 18 years old to participate. Finally, participants were asked whether they were affected by visual or color impairments to ensure they could perceive all aspects of the aesthetic manipulation.

In the second phase, participants were presented with a cover story and a detailed task description (exact wording provided in the Supplementary tables and figures on OSF). Next, participants were randomly assigned to the aesthetic or unaesthetic variant of the app. As a cover story, participants were asked to interact with the web app and review it as part of a usability test, likewise to past research (Hamborg et al., 2014). They were also told that they would have to answer a series of questions about the app's content once they completed their exploration. Here, it was emphasized that a conscientious exploration of the app was necessary to answer the upcoming questions correctly and that they were not allowed to leave the app open while answering the questions. Thus, they received clear goals to fulfill during their interaction with the app (i.e., searching for information on the stimuli website to answer the content questions). By clicking a button, participants were redirected to the app in a new web browser tab and could interact with it at their discretion. It was up to them to decide when to end the exploration and return to the study.

In the third phase of the study, participants answered the six performance questions previously described. Performance time was collected automatically during this process. Afterward, participants filled out the VisAWI and UMUX. The items of each survey scale were presented in randomized order. An attention check item was added among the VisAWI items to ensure adequate data quality ("This is a question to test if you are attentive. Please select (7) strongly agree"). Finally, participants were asked to self-report the quality of their data ("In your honest opinion, did you fill out the survey attentively and should we use your data in our analyses in this study").

In the final phase of the study, participants had the opportunity to provide feedback regarding the survey. Afterward, they received a personalized completion code to claim their compensation through MTurk and were debriefed on the study's purpose. Participants received $2 upon full completion of the study. The OSF repository contains a schematic representation of the study process and a printout of the online survey.^13^

## 4. Results

All analyses were performed using the statistical software R (version 4.2.0, R Core Team, 2022). The level of statistical significance was set at α = 0.05. To investigate possible differences between conditions, we used parametric and non-parametric statistical tests of significance. In case of non-significant results, we further used equivalency tests. In addition, we used bootstrapping to gain further insight into the robustness of our findings. For this, we drew 1,000 data sets from our original data (with replacement), sampling the same amount of participants per condition as in the original data (*n*_*aesthetic*_ = 139, *n*_*unaesthetic*_ = 142). We then calculated *t*-tests for each of the 1,000 data sets. Exact means and standard deviations for all key variables per condition are presented in Table 1, and results from the statistical tests are listed in Table 2.

**Table 1:** Mean, standard deviation and range for key variables sorted by app variant (aesthetic vs. unaesthetic).

	**Aesthetic (*****n*** **= 139)**			**Unaesthetic (*****n*** **= 142)**		
	**Mean**	**SD**	**Range**	**Mean**	**SD**	**Range**
VisAWI—Simplicity	5.67	1.03	2.80–7.00	4.27	1.45	1.20–7.00
VisAWI—Diversity	5.31	1.05	2.20–7.00	4.06	1.62	1.20–7.00
VisAWI—Colorfulness	5.81	1.06	2.00–7.00	3.67	1.83	1.00–7.00
VisAWI—Craftsmanship	5.68	1.13	1.75–7.00	3.99	1.73	1.00–7.00
VisAWI—Total Score	5.62	0.95	2.94–7.00	4.00	1.54	1.23–7.00
UMUX score	80.19	18.47	25.00–100.00	61.44	24.43	4.17–100.00
Performance time (minutes)	2.52	2.03	0.22–14.07	2.65	2.27	0.28–13.32
Performance score	5.26	1.23	0.00–6.00	4.95	1.58	0.00–6.00

SD = standard deviation.

**Table 2:** Results from statistical tests used to compare the two app variants.

**Variable investigated**	**Test used**	**Test statistics**
Perceived aesthetics	Welch's two-sided *t*-test	*t*_(236.20)_ = 10.63, *p* < 0.0001, *d* = 1.26
Perceived aesthetics	Wilcoxon rank sum test	*W* = 15, 877, *p* < 0.0001
Perceived usability	Welch's two-sided *t*-test	*t*_(262.33)_ = 7.26, *p* < 0.0001, *d* = 0.86
Perceived usability	Wilcoxon rank sum test	*W* = 14, 260, *p* < 0.0001
Performance time	Two-sided *t*-test	*t*_(279)_ = −0.52, *p* = 0.60, *d* = −0.06
Performance time	Wilcoxon rank sum test	*W* = 9, 744.5, *p* = 0.86
Performance time	Equivalence test	*t*_(276.74)_ = −0.10, *p* = 0.54
Performance score	Two-sided *t*-test	*t*_(279)_ = 1.82, *p* = 0.07, *d* = 0.22
Performance score	Wilcoxon rank sum test	*W* = 10, 526, *p* = 0.28
Performance score	Equivalence test	*t*_(265.79)_ = 0.99, *p* = 0.84

*d* = Cohen's d for effect size.

### 4.1. Manipulation check: perceived aesthetics

First, the subjective aesthetic perception of the two app versions was investigated using the VisAWI data. This was also seen as a manipulation check, examining whether the participants perceived the aesthetics of the two app variants as intended. Using a Welch's two-sided *t*-test with unequal variances, the aesthetic variant scored significantly higher in the VisAWI total score than the unaesthetic variant. Given the sufficiently large sample size, the *t*-test should still provide reliable results despite a non-normal distribution of the data (Lumley et al., 2002; Bortz and Schuster, 2010). Nevertheless, a Wilcoxon rank sum test was also calculated because equal variances and normal distribution were not given, showing a significant difference between the two groups. Furthermore, the VisAWI total score of the aesthetic variant exceeded the cut-off for an aesthetic interface of 4.5 by Hirschfeld and Thielsch (2015), whereas the unaesthetic variant fell below it. Bootstrapping results showed average values of *t* = 10.72 and *p* < 0.0001, with all 1,000 *t*-tests showing a *p* < 0.05. Out of the 1,000 bootstrapped *p*-values, 527 were equal to or smaller than the value observed with the actual data. Based on these results, we concluded that the manipulation of app aesthetics was successful, given that participants perceived the aesthetic app variant as more aesthetic than the unaesthetic one.

### 4.2. Perceived usability

As discussed in the methods section, only the aesthetics of the two variants of the app were manipulated. Care was taken to keep all other aspects of the apps the same, including avoiding strong manipulations of system usability that have been used in previous studies. Nevertheless, it was expected that users of the aesthetically pleasing variant of the app would exhibit higher levels of subjective usability than users of the unaesthetic one (H1). A comparison of the UMUX ratings for the two variants, using a Welch's two-sided *t*-test with unequal variances, showed that subjective usability was rated significantly different depending on the app's aesthetics. Users of the aesthetic app rated usability significantly higher than those of the unaesthetic variant. A Wilcoxon rank sum test was also calculated because equal variances and normal distribution were not given, showing a significant difference between the two groups. Bootstrapping results for the UMUX showed average values of *t* = 7.36 and *p* < 0.0001, with all 1,000 *t*-tests showing a *p* < 0.05, and 522 *p*-values smaller than or equal to the originally observed value. Results thus favor a robust difference between the two app variants across the 1,000 data sets. This close link between the subjective judgment of aesthetics and perceived usability is consistent with findings from past research (Gu et al., 2016; Minge and Thüring, 2018; Otten et al., 2020) and is in favor of the first hypothesis.

### 4.3. Task performance

The dependent variable performance was operationalized by task performance time and performance score, which we treated separately in the analysis.

#### 4.3.1. Performance time

Regarding the task completion time of the performance tasks, a shorter performance time was expected for the aesthetic variant of the app than the unaesthetic one (H2). A comparison of the performance time for the two variants, using a two-sided *t*-test with equal variances, showed no significant difference between users of the aesthetic app compared to the unaesthetic variant. Because the data were not normally distributed, a Wilcoxon rank sum test was also calculated, showing no significant difference between the two groups.

Given the non-significant difference between the two conditions, we further calculated tests of equivalence (Lakens, 2017; Lakens et al., 2018) to see whether there truly was no meaningful effect or if there was insufficient statistical power to detect the presence or absence of a meaningful effect. Based on the effect from the meta-analysis by Thielsch et al. (2019b, *g* = 0.06), we set the smallest effect size of interest at *d* = 0.05. The equivalence test was non-significant, thus the two groups could not be considered statistically equal. Finally, bootstrapping results showed average values of *t* = −0.47 and *p* = 0.46, with 933 out of 1,000 *t*-tests non-significant and no p-values smaller than or equal to the observed value. From this, we concluded that the groups did not differ significantly regarding the performance time but were also statistically non-equivalent. These results, therefore, argue against the second hypothesis, considering descriptive statistics, the significance tests, and the results from bootstrapping. Only the equivalency test indicated a possible difference.

#### 4.3.2. Performance score

Regarding the performance score, a higher performance score was expected in the aesthetic condition than in the unaesthetic one (H3). A comparison of the performance score for the two variants, using a two-sided *t*-test with equal variances, showed no significant difference in the performance score between users of the aesthetic app compared to the unaesthetic variant. A Wilcoxon rank sum test was also calculated because normal distribution was not given, which showed no significant difference between the two groups.

Because of the non-significant difference, we again performed an equivalence test with a smallest effect size of interest of *d* = 0.10 based on the effect from Thielsch et al. (2019b, *g* = 0.12). The equivalence test was non-significant, indicating that the performance score for the two groups was not equal. The bootstrapping of 1,000 data sets showed an average of *t* = 1.86 and *p* = 0.17, with 449 significant *t*-tests and 526 *p*-values smaller than or equal to the observed value. These results thus provided mixed evidence concerning the third hypothesis that higher app aesthetics improves performance. While results from the *t*-test and the Wilcoxon rank sum test provided evidence against H3, the equivalence test showed that the two groups were not equivalent concerning the performance score. The bootstrapping further revealed that while the average p-value was not significant, almost half of all bootstrapped *t*-tests would be (44.90%).

### 4.4. Correlations among variables

Finally, Pearson's product-moment correlations were calculated to investigate further the relationships among the UMUX score, the VisAWI score, and the performance measures (time and score). Results showed a significant large positive correlation between the UMUX and VisAWI scores [*r*(279) = 0.79, 95% CI (0.74, 0.83), *p* < 0.0001]. There was one additional significant small positive correlations between the performance score and the UMUX score [*r*(279) = 0.23, 95% CI (0.11, 0.33), *p* < 0.001]. All other correlations were non-significant. Table 3 highlights correlations among key variables considered in the present study, and the Supplementary material contain all correlations, including the sub-scales of the VisAWI.

**Table 3:** Correlations among key variables investigated.

	**VisAWI score**	**UMUX score**	**Performance time**
UMUX score	0.79^*^^*^^*^^*^		
Performance time	0.00	–0.10	
Performance score	0.05	0.23^*^^*^^*^	0.10

**** *p* < 0.0001;

*** *p* < 0.001.

## 5. Discussion

The idea that aesthetics has a measurable impact on performance has been the focus of numerous research studies (e.g., Douneva et al., 2016; Gu et al., 2016; Thielsch et al., 2019a; Baughan et al., 2020; Reppa et al., 2021), including a meta-analysis (Thielsch et al., 2019b). However, to the extent of our knowledge, little to no empirical evidence for such an effect exists in the context of smartphone devices. Furthermore, there appears to be no other study investigating the impact of aesthetics on performance that worked with a smartphone app whose actual layout was aesthetically manipulated. Therefore, the present study provides empirical evidence for the influence of aesthetics on performance in the context of smartphone use. Following the call from past research (Thielsch et al., 2015, 2019b), great care was taken to develop both a realistic app and a set of performance tasks for participants' interaction. For this purpose, the aesthetics of a smartphone web app were manipulated to develop two aesthetically different variants of an otherwise identical app. In addition, while the performance questions used were relatively easy, favorable CFA results, high internal consistency, and consistent item analysis metrics show that the items formed a uniform performance measure. We validated all study elements in preliminary discussions to ensure a high transferability of results into practice. Results showed that the two app variants significantly differed in participants' perceived usability and perceived visual aesthetics. No statistically significant differences in performance time or performance score were found. However, equivalency tests also showed that the two groups were not statistically equivalent concerning both performance measures. Furthermore, bootstrapped *t*-tests for the performance score were significant around half of the time (44.90%). These results, alongside the slightly higher performance score in the aesthetic condition, thus point towards an effect of app aesthetics on performance.

### 5.1. Manipulation of app aesthetics

A notable strength of the present study was that the participants interacted with a realistic smartphone web app manipulated in the aesthetics of its user interface. Therefore, participants based their impressions on real interactions rather than mere screenshots or mock-ups. Consequently, the study's effects were found after an actual interaction with a functional smartphone app. The duration of this interaction was not constrained, just as an interaction in everyday life might not be subject to any particular constraints either. To our knowledge, no comparable experimental setup with smartphone apps has been used in past research to study performance in this context. Therefore, the present study extends the existing literature by ensuring that the interaction with a system took place for a longer time and that the system under consideration was an interactive app. This realistic interaction with an app is a crucial addition to the existing literature, as most studies have focused only on screenshots (Thielsch et al., 2015), computer applications (Gu et al., 2016; Otten et al., 2020), or devices manipulated in their external aesthetics rather than the actual interface (Sonderegger and Sauer, 2010; Sonderegger et al., 2014; Minge and Thüring, 2018).

The manipulation of aesthetics used in this study resulted in a significant difference between the two app variants and a large effect of said manipulation on participant's perceived aesthetics (*d* = 1.26, Cohen, 1988). Therefore, the results of this study provide evidence that the chosen manipulation of aesthetics, based on the findings of Seckler et al. (2015) and the definition of aesthetics by Moshagen and Thielsch (2010), is effective in the context of smartphone apps. The present findings further indicate that the results from Seckler et al. (2015) initially found in a desktop computer context are transferable to mobile devices. This effect of the aesthetics manipulation implies that design aesthetics play a similar role in the context of mobile smartphone devices regarding the user's subjective perception of aesthetics compared to desktop computers. Considering that design is constantly evolving, and people's perceptions and tastes change over the years (Ntoulas et al., 2004), the findings from the present study further show that results from several years ago can still be applied to current applications. The present study's findings thereby provide guidance for professionals in research and industry concerning the aesthetics of digital applications.

### 5.2. Perceived aesthetics and usability

Although we took care to manipulate the two app variants solely in their aesthetics, participants interacting with the aesthetic variant of the app rated it as significantly more usable after the interaction, showing a large effect of the aesthetics manipulation on perceived usability (*d* = 0.86). Thus, results favor the first hypothesis that users of the aesthetic variant experienced significantly higher subjective usability than users of the unaesthetic one (H1). This finding is consistent with past research (Moshagen et al., 2009; Sonderegger and Sauer, 2010; Sonderegger et al., 2014; Gu et al., 2016; Minge and Thüring, 2018; Otten et al., 2020; Schrepp et al., 2021). Consequently, this study provides further evidence for aesthetics' effect on perceived usability, expanding past evidence to the context of smartphone web apps. One explanation for these results is a so-called halo effect of the aesthetics manipulation on perceived usability, which has been discussed in past research (Tractinsky et al., 2000). Applied to the results found here, it postulates that the high aesthetics of the app implies high subjective usability. As a result, the participants perceive higher subjective usability, although both variants are objectively the same. The present study hence provides evidence that such a halo effect between aesthetics and usability exists not only in a desktop computer context but also in the context of smartphones.

### 5.3. The effect of aesthetics on performance

Numerous studies have already explored the interaction of aesthetics and performance (e.g., Sauer and Sonderegger, 2011; Sonderegger et al., 2014; Douneva et al., 2016; Gu et al., 2016; Thielsch et al., 2019a; Baughan et al., 2020; Reppa et al., 2021). Despite this, there is still no consensus on whether aesthetics affect performance, as research findings so far have been too ambivalent (Thielsch and Niesenhaus, 2017). This is especially the case for smartphone devices, where there is still little to no research that addresses the aesthetics of the actual user interface of smartphone apps and their effects on performance.

#### 5.3.1. Performance time

Concerning the effect of aesthetics on performance time, results did not reveal a significant difference between the two conditions, consequently leading to the rejection of hypothesis two (H2, shorter performance time for the aesthetic app variant compared to the unaesthetic app). While the two groups were also statistically non-equivalent, results from the bootstrapping showed no significant difference in most cases (93.30%). These findings correspond to the results of Thielsch et al. (2019a), who also found no significant effect of aesthetics on performance time. A possible explanation for this non-significant difference could be that participants did not have a time limit to complete their task in the present study. Thus, the factor time might not have been relevant for the participants, leading to an absence of time pressure, causing the app exploration to take about the same amount of time for participants in both conditions. On the other hand, the present study worked with a crowd-sourced sample from MTurk, where participants are likely to be pressured to complete as many tasks in as little time as possible to increase their payment. Therefore, time might have played a similar and essential role for participants in both conditions. Furthermore, there was substantial variability in performance time across participants in both groups. Given that the online survey platform automatically collected the time participants spent on the survey page containing the performance questions, we could not monitor participants' actual behavior during this time. It is thus possible that some participants had the performance questions open during exploration despite instructions telling them not to, leading to a longer performance time. Others who followed the instructions likely had shorter performance times, reflecting the time spent just answering the questions without the exploration. This limitation of the performance time variable has to be kept in mind when interpreting the results, although the issue was presumably present in both conditions.

#### 5.3.2. Task performance

The present work provides mixed evidence concerning the effect of aesthetics on user performance. Using a set of self-developed questions, summarized in a performance score, results revealed a small but non-significant effect of aesthetics on performance (*d* = 0.22), comparable to the effect reported in the meta-analysis by Thielsch et al. (2019b, *g* = 0.12). This agreement regarding a small effect strengthens the assumption that app interface aesthetics affect performance. However, results showed no statistically significant difference between conditions. Still, while we found no significant difference, we also found no statistical equivalence between the two groups. Taken alongside the descriptively higher performance score for the aesthetic condition and the results from bootstrapping, our findings point toward an effect of app aesthetics on user performance. Results thus indicate that participants might perform significantly better with an app's aesthetic variant than with the unaesthetic one, which favors hypothesis three (H3, higher performance expected for users interacting with the aesthetic app compared to the unaesthetic variant).

Several reasons might explain the absence of a statistically significant difference in performance in the present study. First, most participants answered the questions correctly, given the high average performance scores in both conditions. Thus, they might have already had the questions open while exploring the app, despite the instructions telling them otherwise. This behavior might have influenced participants' performance in both conditions, causing performance to be better than initially expected. Second, the combination of both non-significant null hypotheses significance tests and equivalency tests indicates that the study might have been statistically underpowered to investigate the presence or absence of a meaningful effect thoroughly (Lakens et al., 2018). Results from bootstrapping further undermine this point, with around half of all bootstrapped *t*-tests significant. Thus, larger samples are needed in future studies investigating the effect of app aesthetics on performance. Given the limited number of studies on the effects of aesthetics on performance in the smartphone context, the current study's results thus provide initial evidence for this effect. Third, users' motivations also feasibly influence performance. In the present study, completion time likely was more important to participants than correctly following the task instructions and answering the questions, given the crowd-sourced sample. Nevertheless, the fact that most questions were answered correctly by participants in both conditions argues against this assumption. While the present work did not consider users' motivation as a confounding factor for performance, future work should.

The results of the present study suggest that the aesthetics of a web app can affect users' performance to a similar extent as what was previously found in other contexts. Thielsch et al. (2019b) concluded that aesthetics significantly affected performance with mobile devices (e.g., non-smartphone cell phones) and software applications, but not on websites. The present study thus contributes to these findings, showing that app aesthetics has the potential to affect user performance, although further investigation is needed.

### 5.4. Implications of results

In summary, the present results provide evidence regarding app aesthetics' effect on subjective (perceived aesthetics and usability) and objective (performance time and score) elements of a user's interaction with a smartphone app. While results indicate no or mixed effects on performance, they suggest an apparent effect of aesthetics on users' subjective experience. While such effects have been found in past research, studies in a smartphone context are still limited. The present study thus is among the first to show that close links between objective aesthetics and subjective perceptions of a system exist within a smartphone context. Even if one assumes that aesthetics do not affect performance in a smartphone context, they have apparent effects on the users' subjective perception. Considering that the subjective perception of the app (i.e., aesthetics, usability) differed significantly between conditions, results highlight that while users do not take less time to complete a task with an aesthetic website, they definitely have an improved subjective experience while arguably performing better.

#### 5.4.1. Theoretical explanations

Regarding past explanations from related work, the results do not support any existing ideas concerning aesthetics' effects on performance. For instance, the significantly higher perceived usability and the slightly better performance score in the aesthetic condition speak for the presence of attentional and cognitive effects (Szabo and Kanuka, 1999; Tractinsky et al., 2000). According to this notion, a more aesthetic design would promote the automatic processing of information, thereby increasing performance, which would explain the somewhat better performance score in the aesthetic condition. However, attentional and cognitive effects can not explain why the evaluation of performance time did not reveal any significant differences, given that faster performance times in the aesthetic condition would also be expected. As described above, the halo effect could explain the differences in subjectively perceived higher usability, although performance differences are unrelated to this effect. At the very least, however, it can be stated that the results of this study argue against the prolongation of joyful experience theory (Sonderegger and Sauer, 2010; Sonderegger et al., 2014). The performance times of the two groups did not differ significantly and did not indicate a prolonged exploration of the aesthetic variant, although the MTurk setting likely influenced these results. Therefore, based on the present results, only conjectures can be made regarding theoretical rationales.

#### 5.4.2. How to study performance

The disparate effects of aesthetics on performance highlight the importance of carefully considering how performance can be operationalized. In the present study, we worked with two ways to quantify users' performance: a self-developed set of content-related questions and the time taken to fill out those questions. While the aesthetic manipulation did not affect performance time, we found mixed results for the performance questions, which suggests that aesthetics affect performance differently depending on the chosen performance indicator.

First, this raises the question of what we denote when discussing performance. While completing a task quickly and efficiently might be crucial in some cases, error-free task completion is of greater importance in others. In the present study, our approach focused on the correct gathering of information to answer specific questions while also considering the time taken for this information-gathering. Thus, high performance meant that users processed and recalled information better (i.e., higher performance score) and faster (i.e., shorter performance time). We thus considered performance from two perspectives.

Second, researchers need to think about how they can measure performance. Standardized scales, such as those used for measuring subjective aesthetics and usability, make little sense for performance, given the high context-bound nature of possible tasks. For the present study, we designed questions to measure performance close to real life, but measuring performance has different approaches. In our study, performance was related to the site's content, which is not always the case. Other approaches include the number of errors, number of commands, or the amount of additional information needed for task completion (Thielsch et al., 2019b). When looking at the data from our performance score, we see a ceiling effect, with most participants getting the majority of questions correct. The choice of performance measure thus influenced our results. Different methods for measuring performance will likely highlight different effects that interface aesthetics and other design factors can have on users.

Thus, researchers should consider different ways of operationalizing performance with mobile devices beyond those used in the present study (i.e., number of correct answers, task duration). Future research comparing different performance measures in varying contexts could deliver additional insight into the effects of aesthetics on user performance. Furthermore, the boundaries of these effects should be explored by using a variety of tasks, more questions, or questions with more considerable differences in difficulty.

#### 5.4.3. How to define aesthetics

Another plausible explanation for the disparate results on the relationship between aesthetics and performance, both in the present paper and in past research, is the multi-factorial construct of aesthetics itself. It is conceivable that different facets of aesthetics have distinct effects on performance and therefore require specific explanations for the individual facets. For example, while the color of an app might impact performance, symmetry might not (or vice versa). Within HCI research, there is still no uniform definition of aesthetics, and research studies sometimes show imprecise or even missing definitions of the examined constructs (Thielsch et al., 2019b). A lack of shared definitions complicates the comparability and interpretation of results across research immensely (Flake and Fried, 2020) and could also explain the contradictory results regarding the effect of aesthetics on performance. In the present work, we only had two app variants manipulated in terms of overall aesthetics. App variants with differences in only certain facets of aesthetics could provide further insight. Future research should thus address these questions and investigate the effects of different facets of aesthetics, mentioned in definitions, on performance.

#### 5.4.4. How to measure aesthetics

In line with the question of how to define aesthetics comes the issue of how to measure it. Just as with definitions, there is a lack of common standard regarding how aesthetics is measured (Thielsch et al., 2019b). Hassenzahl and Monk (2010) argued that contradictory results on the effects between usability and aesthetics could be due to different measurement methods. This likely is also the case for aesthetics and performance. Thielsch et al. (2019b) in their meta-analysis looked at methods used to measure aesthetics and found that many researchers rely on unstandardized measures with varying levels of psychometric quality. Furthermore, using unstandardized measures was associated with larger effects than standardized aesthetics measures such as the VisAWI or the scale by Lavie and Tractinsky (2004). Thus, the varying methods used to measure aesthetics further explain the contradictory results in past research. In addition, given that survey scales are based on underlying theoretical models, these models need to be made clear and investigated whenever one uses survey scales for measurement (DeVellis, 2017; Flake and Fried, 2020). However, investigating the factor structure of the VisAWI raised doubt about the current model used for the scale. As briefly mentioned in the methods section, our attempts to confirm the factor structure of the VisAWI were unsuccessful, leading us only to consider the rating of overall perceived aesthetics. These doubts not only limited our possibilities to investigate the effect that different facets of the app aesthetics have on performance but also challenged the underlying theory behind the VisAWI and the understanding of aesthetics by Moshagen and Thielsch (2010). However, neither the theoretical structure nor the psychometric quality of the VisAWI was the focus of the present study. Future research on both the quality of the scales used within aesthetics research and the theoretical models behind them is thus needed.

#### 5.4.5. Practical implications

Past research has shown that aesthetics are a way to stand out in a crowded market, increasing recognition value and thus making pleasing aesthetics a decisive success factor (Bloch et al., 2003; Bhandari et al., 2015, 2019). However, previous work has investigated aesthetics mainly outside the context of smartphone apps. The present study thus extends past findings, showing that users perceive an aesthetic app as more aesthetic and more usable. Furthermore, aesthetics appear to impact user performance, although to a lesser extent. Designers need to be aware of these effects when working on their products. An app with good aesthetics is more attractive to users, possibly causing them to use the app more, even if they perform equally independently of the app's aesthetics. While some have expressed fears in the past regarding a possible negative impact of aesthetics on performance (e.g., Andre and Wickens, 1995), results from the present study further ease these worries. At the very least, aesthetics do not negatively affect user performance but might positively impact it while definitely influencing the user's subjective experience. On top of the effects found in the present study, there are additional consequences of aesthetics already shown in previous studies. Higher user preference, trust, satisfaction, and willingness to reuse are all related to pleasing aesthetics (Moshagen and Thielsch, 2010). Practitioners should always keep this in mind when considering which aspects of software development are most important. Based on this work's findings, it is clear that investing in the design of the interface and placing great emphasis on aesthetic design is worth it.

### 5.5. Limitations and future research

The first limitation of this work was that the app to interact with was a web app. Using a web app allowed us to distribute our stimuli to participants regardless of their operating system, with no need for participants to install the app. However, differences between web and native apps might have affected the results. Although the editor used to create the app variants was comparable in features and behavior to a native app (Jobe, 2013), readers should note that no native app was used in this study. Future work should thus replicate this study with native apps.

In addition, this study did not collect information about the use context. Several papers (e.g., van Schaik and Ling, 2009; Sonderegger and Sauer, 2010; Iten et al., 2018) mentioned that the positive effect of aesthetics on performance tends to manifest in a work context. Thus, a system's use context may impact the aesthetics' effect on performance, which should be considered in future research. However, because the present study used crowd-sourcing workers, participants were arguably within a work context mindset.

Third, although the present study used an interactive product (instead of just screenshots), the average duration of interaction was still relatively short (given the overall study duration). The present study thus focused mainly on the users' experience during or directly after the interaction while not looking at other relevant time frames, such as the users' experience before the interaction, afterward, or over time. Further investigation during different time points in the users' interaction cycle would allow for a better understanding of whether and how perceived usability and performance change due to interacting with an aesthetically manipulated app and whether the found effects are stable over time.

Fourth, given the MTurk sample, participants were likely not overly interested in exploring the stimuli app in detail but wanted to complete the study as fast as possible. Given that our performance measures were not directly related to workers completing their task on MTurk, and thus receiving their payment, motivation to respond to the performance questions correctly was likely limited. Still, past research has shown that MTurk samples are comparable in quality to other more traditional online samples while demographically more diverse (Buhrmester et al., 2011).

Next, the screening of participants concerning visual and color impairments was based exclusively on self-reporting. It can, therefore, not be ruled out that some participants affected by these types of impairments took part in the study. Future studies should anticipate this and integrate a color and vision test to ensure that all aspects of the aesthetic manipulation are perceived as intended.

Finally, the present paper focused on aesthetics' effects on performance. Therefore, for successful manipulation of aesthetics, the differences between the app's aesthetic and unaesthetic variants were as extensive as possible. Given that the difference in aesthetics between the two variants of the app was rather extreme, future work could look at different levels of aesthetics and find out where the thresholds are for both differences in subjective experience and user performance. Similarly, only two app variants were investigated without detailed differentiation on the level of individual facets of aesthetics. Thus, no conclusions could be drawn as to which facets contributed to the changed performance and perception. Follow-up studies should investigate which aesthetic aspects lead to performance changes, allowing researchers and professionals to draw conclusions for their work and adapt their aesthetic concepts accordingly.

## 6. Conclusion

The smartphone industry represents a vast market with seemingly endless potential. However, the specifics of smartphone interfaces and their applications have not yet been sufficiently researched to adequately understand user behavior and experience. Specifically, the aesthetics of apps and their effects on users' subjective experience and performance have seen little research in the past. This paper represents a first attempt to investigate the influence of aesthetics on performance in the context of a functional smartphone app. Two variants of a web app were created, manipulated only in terms of aesthetics. Participants in an online study (*N* = 281) were asked to interact with one of the two app variants before answering content-related questions and filling out standardized survey scales on perceived usability and aesthetics. Results showed that the aesthetically pleasing app variant led to a significantly higher perception of aesthetics and usability. Furthermore, the results point toward an effect of aesthetics on performance, with participants interacting with the aesthetic variant exhibiting slightly better performance. Based on this study, it can be concluded that aesthetic smartphone apps not only look nicer but also have the potential to boost performance. Aesthetics is more than just a "nice to have" feature and represents an essential aspect of applications that should always be considered.

## Data availability statement

The original contributions presented in the study are included in the article/Supplementary material, further inquiries can be directed to the corresponding author. All supplementary material for this article can be accessed on the Open Science Framework: https://osf.io/qevpk/.

## Ethics statement

Ethical review and approval was not required for the study on human participants in accordance with the local legislation and institutional requirements. The patients/participants provided their written informed consent to participate in this study.

## Author contributions

DU and FB implemented the online study. DU collected the data with the support of SP and wrote the first draft. SP, DU, and FB performed the statistical analysis. SP wrote the second draft of the manuscript. All authors contributed to the conception, design of the study, manuscript revision, read, and approved the submitted version.

## Footnotes

^1^ https://www.merriam-webster.com/dictionary/app

^2^ https://www.merriam-webster.com/dictionary/application

^3^ https://www.youtube.com/watch?v=GSiUzuB-PoI

^4^ https://www.xbox.com/en-US/xbox-game-pass/cloud-gaming

^5^In Psychology, the halo effect refers to a phenomenon where certain characteristics, such as physical beauty, are perceived early in an interaction, consecutively influencing the perception of other personal characteristics (Thorndike, 1920; Dion et al., 1972).

^6^ https://www.mturk.com

^7^ https://wix.com

^8^The German version of the VisAWI-S was used in the preliminary investigation because the participants were German-speaking. However, the app screenshots rated were in English because they were designed to be used with English-speaking participants in the main study.

^9^ https://osf.io/xsdqy

^10^ https://osf.io/amvsk

^11^*CFI* = Comparative Fit Index; *SRMR* = Standardized Root Mean Square Residual; *RMSEA* = Root Mean Square Error of Approximation. The following criteria were seen as an indication of good model fit: Low χ^2^ value and *p*>0.05 for the Chi-squared test, *RMSEA* < 0.06, *SRMR* ≤ 0.08 and 0.95 ≤ *CFI* ≤ 1 (Hu and Bentler, 1999).

^12^ https://www.unipark.com

^13^ https://osf.io/udjkm
